# Short-term and long-term increased mortality in elderly patients with burn injury: a national longitudinal cohort study

**DOI:** 10.1186/s12877-022-03669-1

**Published:** 2023-01-17

**Authors:** Charlotte I. Cords, Margriet E. van Baar, Anouk Pijpe, Marianne K. Nieuwenhuis, Eelke Bosma, Michael H. J. Verhofstad, Cornelis H. van der Vlies, FRAIL group, G. Roukema, Y. Lucas, K. Gardien, E. Middelkoop, S. Polinder, S. M. H. J. Scholten, J. Damen, K. Boudestein, P. P. M. van Zuijlen, F. U. S. Mattace-Raso, Dutch Burn Repository group, A. Boekelaar, D. Roodbergen, M. M. Stoop, P. P. M. van Zuijlen, Y. Lucas, A. van Es, H. Eshuis, J. Hiddingh, S. M. H. J. Scholten-Jaegers, E. Middelkoop, A. Novin

**Affiliations:** 1 Association of Dutch Burn Centres, Maasstad Hospital, Rotterdam, the Netherlands; 2 Trauma Research Unit, Department of Surgery, Erasmus MC, University Medical Center Rotterdam, Rotterdam, the Netherlands; 3 Department of Public Health, Erasmus MC, University Medical Centre Rotterdam, Rotterdam, the Netherlands; 4 Association of Dutch Burn Centres, Red Cross Hospital, Beverwijk, the Netherlands; 5 Department of Plastic Reconstructive and Hand Surgery, Amsterdam Movement Sciences (AMS) Institute, Amsterdam UMC, Location VUmc, Amsterdam, the Netherlands; 6 Association of Dutch Burn Centres, Martini Hospital, Groningen, the Netherlands; 7 Hanze University of Applied Sciences, Research Group Healthy Ageing, Allied Health Care and Nursing, Groningen, the Netherlands; 8 Department of Human Movement Sciences, University of Groningen, University Medical Centre Groningen, Groningen, the Netherlands; 9 Department of Trauma and Burn Surgery, Maasstad Hospital, Rotterdam, the Netherlands

**Keywords:** Burn injury, Five-year mortality, Elderly, Long-term outcome

## Abstract

**Background:**

The population of elderly patients with burn injuries is growing. Insight into long-term mortality rates of elderly after burn injury and predictors affecting outcome is limited. This study aimed to provide this information.

**Methods:**

A multicentre observational retrospective cohort study was conducted in all three Dutch burn centres. Patients aged ≥65 years, admitted with burn injuries between 2009 and 2018, were included. Data were retrieved from electronic patient records and the Dutch Burn Repository R3. Mortality rates and standardized mortality ratios (SMRs) were calculated. Multivariable logistic regression was used to assess predictors for in-hospital mortality and mortality after discharge at 1 year and five-year. Survival analysis was used to assess predictors of five-year mortality.

**Results:**

In total, 682/771 admitted patients were discharged. One-year and five-year mortality rates were 8.1 and 23.4%. The SMRs were 1.9(95%CI 1.5–2.5) and 1.4(95%CI 1.2–1.6), respectively. The SMRs were highest in patients aged 75–80 years at 1 year (SMRs 2.7, 95%CI 1.82–3.87) and five-year in patients aged 65–74 years (SMRs 10.1, 95%CI 7.7–13.0). Independent predictors for mortality at 1 year after discharge were higher age (OR 1.1, 95%CI 1.0–1.1), severe comorbidity, (ASA-score ≥ 3) (OR 4.8, 95%CI 2.3–9.7), and a non-home discharge location (OR 2.0, 95%CI 1.1–3.8). The relative risk of dying up to five-year was increased by age (HR 1.1, 95%CI 1.0–1.1), severe comorbidity (HR 2.3, 95%CI 1.6–3.5), and non-home discharge location (HR 2.1, 95%CI 1.4–3.2).

**Conclusion:**

Long-term mortality until five-year after burn injury was higher than the age and sex-matched general Dutch population, and predicted by higher age, severe comorbidity, and a non-home discharge destination. Next to pre-injury characteristics, potential long-lasting systemic consequences on biological mechanisms following burn injuries probably play a role in increased mortality. Decreased health status makes patients more prone to burn injuries, leading to early death.

## Introduction

Burn injury is the fourth most common trauma mechanism worldwide [1, 2]. A recent Dutch study revealed that around one-fifth of adult burn injury-related admissions concerned elderly patients i.e., aged 65 years and older [3]. Research conducted by the Dutch government showed that the elderly population lives independently at home longer with an increasing mean age [4]. As a consequence, it is most likely that the overall incidence of burns in the elderly population will increase accordingly [5]. The elderly burn population constitutes a vulnerable and often challenging group for specialized burn care [6, 7]. Elderly can be particularly prone to burn injury due to impaired judgement, coordination, balance, and reaction time. This may make them less able to escape harm [1, 8]. Furthermore, thinning skin and decreased skin sensation contribute to an increased risk of burns when exposed to heat [6, 9].

Despite the challenging nature of the treatment of elderly patients with burn injuries, an extensive improvement in short-term outcomes has been established in the last decade, showing a substantial decrease in in-hospital mortality [9–11]. Nevertheless, recovery after burns in elderly patients is often still poor [5, 6, 10, 12], and in-hospital mortality is high compared to other age groups [9, 10, 13]. Known risk factors for in-hospital mortality in elderly burn patients are burn size, inhalation injury, revised Baux score, comorbidity, and age [7, 9, 14].

Unlike these well-known predictors of short-term outcomes, contradictory findings have been reported about long-term mortality in elderly and the association to burn injury [9, 15]. Some studies observed no correlation between burn injury and long-term mortality in elderly [16], while others did [14, 17]. This difference is probably related to the heterogeneity in those study populations and because they do not assess short-term and long-term mortality in one study. Therefore, the primary aim of this study was to assess long-term mortality at 1 year and 5 years after discharge in elderly patients with burn injury and a primary admission to a Dutch dedicated burn care centre. Furthermore, short-term mortality, i.e., in-hospital mortality, was assessed. Lastly, predictors for long-term mortality were determined and compared to in-hospital mortality predictors.

## Methods

### Study design and population

A multicentre observational retrospective cohort study was conducted in all Dutch dedicated burn centres (Red Cross Hospital, Beverwijk; Martini Hospital, Groningen and Maasstad Hospital, Rotterdam). All patients aged 65 years and over, admitted ≥2 hours with burns between 2009 and 2018, were eligible for inclusion. The study protocol was not subject to Medical Research involving Human Subjects Act and was approved by the local review boards.

### Data collection

Data were derived from the national burn registry of the three burn centres in the Netherlands (Dutch Burn Repository R3), which started collecting data from 2009 onwards. This database is filled by dedicated burn care professionals, where quality monitoring and improvement is formally organized. Data on patient characteristics, burn characteristics, as well as treatment characteristics and in-hospital mortality, are documented (Table 1). Comorbidity according to the American Society of Anaesthesiologists classification (ASA), was derived from the electronic patient files or assessed by reviewing the patient history documentation. Data on mortality status was obtained from the electronic patient file or checked based on a national insurance database (VECOZO system) or The Municipal Personal Data Administration (GBA check). Socio-economic status was based on work participation, income, and education found in patients’ postal code areas following the method of The Netherlands Institute for Social Research and converted into so-called status scores [18]. These status scores were classified into quintiles (1 = lowest, 5 = highest), and the lowest quintile was considered low socio-economic status.

**Table 1:** Baseline characteristics of all admitted patients vs discharged patients

	Admitted patients, n (%) (***n*** = 771)	Discharged patients, n (%) (***n*** = 682)
**Characteristics**		
Sex: female (%)	371 (48.1)	322 (47.2)
Age, years (%)		
65–74	390 (50.6)	364 (53.4)
70–84	253 (32.8)	224 (32.8)
≥ 85	128 (16.6)	94 (13.8)
Socio-economic status^1,2^ (%)		
1	223 (29.7)	197 (29.7)
≥ 2	527 (70.3)	467 (70.3)
ASA Score^1,2^ (%)		
1–2	423 (57.4)	407 (61.8)
≥ 3	314 (42.6)	252 (38.2)
Aetiology^1,2^ (%)		
Flame	415 (54.1)	343 (50.4)
Scald	181 (23.5)	169 (25.0)
Contact	86 (11.2)	83 (12.3)
Fat	32 (4.2)	32 (4.7)
Chemical	22 (2.9)	22 (3.4)
Other^3^	30 (4.1)	22 (3.4)
TBSA (%)		
< 5	441 (57.2)	428 (62.8)
5–20	245 (31.8)	220 (32.3)
> 20	85 (11.0)	34 (5.0)
Surgery (%)	541 (70.2)	506 (74.2)
TBSA excision (%)		
No surgery	230 (29.8)	176 (25.8)
< 5%	387 (50.2)	369 (54.1)
> 5%	154 (20.0)	137 (20.1)
Revised Baux Score	81.4 (74.0–91.0)	80.0 (73.3–88.0)
Length of stay (days)	12.0 (2.0–25.0)	14.5 (3.0–26.0)
ICU stay (%)	204 (26.5)	132 (19.4)
Mechanical ventilation (%)	132 (17.1)	75 (11.0)
Discharge location^1,2^ (%)		
Home	483 (71.2)	482 (71.2)
Non-home^4^	195 (28.8)	195 (28.8)

All data are presented as median (P25-P75) or as n (%)

^1^Missing values admitted patients; socio-economic status (*n* = 21), ASA score (*n* = 34), atiology (*n* = 5), discharge location (*n* = 93)

^2^Missing values discharged patients; socio-economic status (*n* = 18), ASA score (*n* = 23), aatiology (*n* = 5), discharge location (*n* = 5))

^3^Including steam, lightning, electricity and “other reasons”

^4^Including nursing facility (*n* = 105), other hospital (*n* = 32), psychiatric facility (*n* = 17), rehabilitation facility (*n* = 10)

### Data analysis

Outcomes were reported as percentages for categorical variables. Continuous variables were summarized as either means with corresponding standard deviations (SD) or medians with 25th -75th percentiles, depending on normality of distribution. Standardized Mortality Ratios (SMRs) were assessed, to gain insight into the observed versus expected mortality rates of the study population. The SMR is the ratio between the observed number of deaths in our study population over a given period to the number that would be expected over the same period if our study population had the same age/sex specific mortality rates as the general Dutch population [19, 20]. The data on mortality of the Dutch population were available on StatLine, Statistics Netherlands [21]. The SMRs with 95% confidence intervals (CIs) were calculated at one-year and 5 years after discharge for the total population and subgroups with the Mid-P exact test using Miettinen’s (1974) modification as described in Epidemiologic Analysis with a Programmable Calculator [22]. Mortality rate per 10.000 person-years at one and 5 years was calculated by dividing the observed number of deaths by the total number of person-years in the follow-up.

Univariable and multivariable logistic regression analysis (backward stepwise LR) was done to identify predictors of in-hospital mortality, and mortality at one-year and between one-year to five-year after discharge. Relative risks were estimated by odds ratios (ORs) with 95% confidence intervals (CIs). For the multivariable analyses, variables were checked for multi-collinearity (Spearman’s r > 0.75). A *p*-value of < 0.20 from the univariable regression analyses was considered to reflect an association between a variable and mortality. Multivariable analyses were performed with a minimum of 10 cases for every estimated parameter. In addition, Cox proportional hazards regression was used to assess risk factors of 5-year mortality, resulting in hazard ratios (HRs) with 95% confidence intervals (CI). Left censoring was applied by excluding patients that died in hospital. Proportional Hazard assumptions were tested by visual inspection of log-log plots of survival and performing hypothesis tests on the Schoenfeld residuals.

Two-tailed *p* values < 0.05 were considered statistically significant for all statistical tests. Data were analysed using IBM SPSS Statistics for Windows version 26 (IBM Corp., NY, USA) and Stata was used to asses Proportional Hazard assumptions.

## Results

### Inclusion

**Fig. 1:**
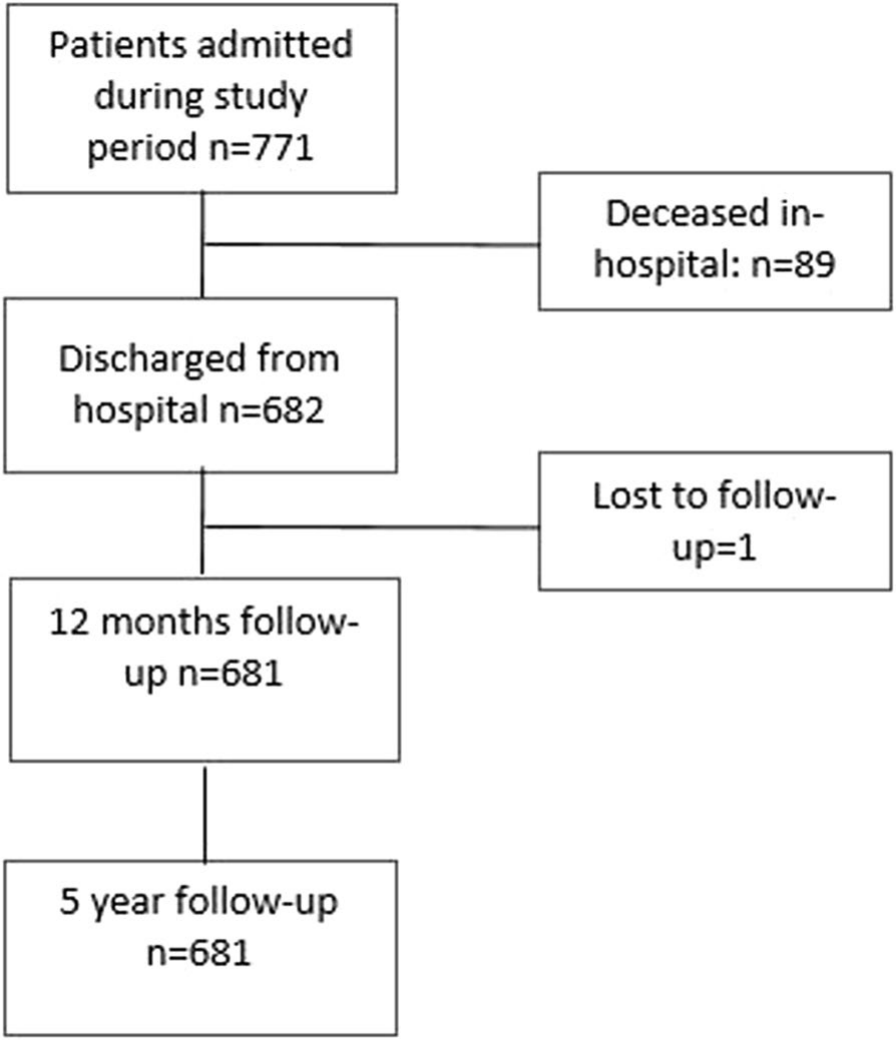
Eligibility chart

A total of 771 patients aged ≥65 years had been admitted, and 682 patients were discharged alive from the Dutch dedicated burn centers with burn injuries between 2009 and 2018 (Fig. 1). Patient and injury characteristics per group are shown in Table 1. Of all admitted patients, 48% were female, the median age was 74 years (25th -75th percentiles 69–81), and the median percentage Total Body Surface Area (TBSA) burned was 4% (25th -75th percentiles 1–9).

### In-hospital mortality

In total, 12% (*n* = 89/771) of all admitted patients died during hospital stay, *n* = 17 patients were moribund. Table 2 shows the univariable and multivariable predictors for in-hospital mortality, median length of stay was 2 days (IQR 1–15 days). Significant independent predictors for in-hospital mortality were severe comorbidity reflected as an ASA-score ≥ 3 (OR 3.8, 95% CI 1.8–8.2), a higher revised Baux score (OR 1.1, 95% CI 1.0–1.1) and admission to the intensive care unit (ICU) (OR 4.9, 95% CI 2.2–10.8). Age, percentage Total Body Surface Area (TBSA) burned, percentage TBSA excised, and mechanical ventilation were not statistically significantly associated with in-hospital mortality in multivariable regression.

**Table 2:** Predictors of in-hospital mortality

	In-hospital mortality (***n*** = 771)^1^		Univariable regression	Multivariable regression^**4**^
Characteristics	Survivors (*n* = 682)	Non-survivors (*n* = 89)	*OR (95% CI)*	*OR (95% CI)*
Sex, Female	322 (47.2)	49 (55.1)	1.37 (0.88–2.14)	
Age, years	74.0 (69.0–80.0)	81 (73.5–88.0)	**1.08 (1.05–1.11)**^**2**^	–
ASA score^3^				
1–2	407 (61.8)	16 (20.5)	1	
≥ 3	252 (38.2)	62 (79.5)	**6.26 (3.53–11.04)**^**2**^	**3.84 (1.81–8.16)**^**2**^
SES score^3^				
Other	467 (70.3)	60 (69.8)	1	
Low SES (1st quintile)	197 (29.7)	26 (30.2)	0.97 (0.60–1.59)	
TBSA burned^5^				
< 5%	428 (62.8)	13 (14.6)	1	
5–19%	220 (32.3)	25 (28.1)	**3.74 (1.87–7.46)**^**2**^	–
≥ 20%	34 (5.0)	51 (57.3)	**49.39 (24.48–99.65)**^**2**^	–
Revised Baux Score	80.0 (73.5–88.0)	112.0 (94.0–127.0)	**1.14 (1.11–1.16)**^**2**^	**1.10 (1.07–1.14)**^**2**^
Surgery				
No	176 (25.8)	54 (60.7)	1	
Yes	506 (74.2)	35 (39.3)	**0.23 (0.14–0.36)**	–
TBSA excision				
No surgery	176 (25.8)	54 (60.7)	1	
< 5%	369 (54.1)	18 (20.2)	**0.16 (0.09–0.28)**^**2**^	–
≥ 5%	137 (20.1)	17 (19.1)	**0.40 (0.22–0.72)**^**2**^	–
Length of stay (days)	14.5 (3.0–26.0)	2 (1.0–15.5)	**0.98 (0.96–0.99)**^**2**^	**0.97 (0.95–0.98)**^**2**^
ICU stay				
No	550 (80.6)	17 (19.1)	1	
Yes	132 (19.4)	72 (80.9)	**17.65 (10.06–30.94)**^**2**^	**4.90 (2.23–10.78)**^**2**^
Mechanical ventilation				
No	607 (89.0)	32 (36.0)	1	
Yes	75 (11.0)	57 (64.0)	**14.42 (8.79–23.65)**^**2**^	–

All data are presented as median (P25-P75) or as n (%)

^1^n represents the number of patients from whom data were available

^2^*p* < 0.05

^3^Missing: ASA *n* = 34, SES *n* = 21

^4^Nagelkerke *r*^2^ = 0.575

^5^Total Body surface area (TBSA). Percentage non-survivors in < 5% group: 2.9%, in 5–19% group: 10.2%, in ≥20% group: 60.3%

### Mortality at one year after discharge

**Fig. 2:**
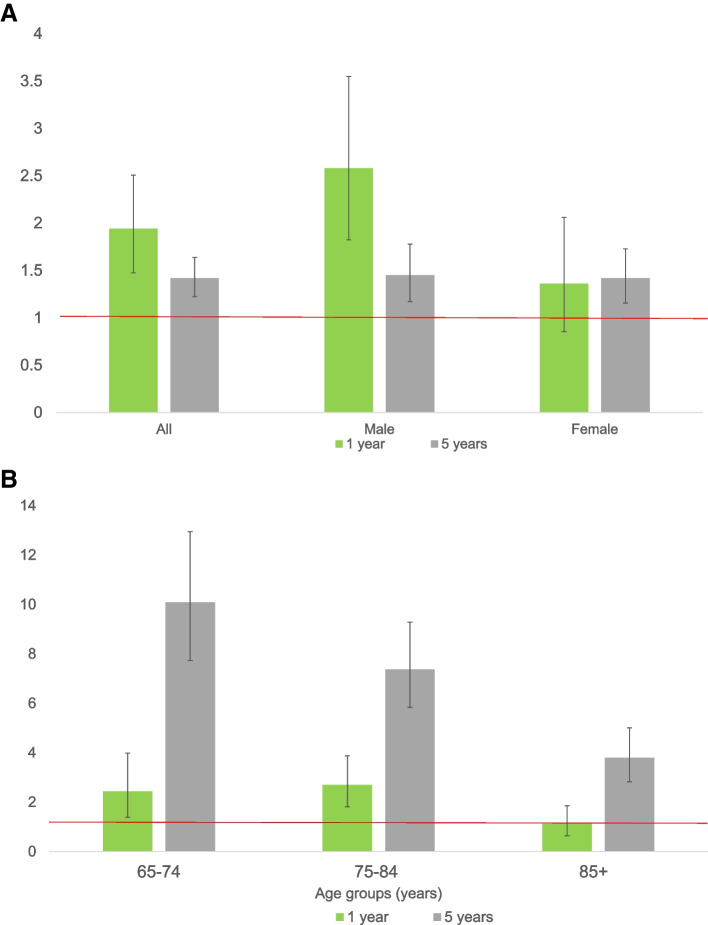
**A** Standardized Mortality Ratios, by sex. One year; SMR all: 1.9 (95% CI 1.5–2.5), SMR Male: 2.6 (95% CI 1.8–3.6), SMR Female: 1.4 (95% CI 0.9–2.1). Five years; SMR all: 1.4 (95% CI 1.2–1.6), SMR Male: 1.4 (95% CI 1.2–1.8) SMR Female: 1.4 (95% CI 1.3–1.7). Red line: SMR 1. **B** Standardized Mortality Ratios, by age**.** One year; SMR 65–74 yrs.: 2.4 (95% CI 1.4–4.0), SMR 75-84 yrs.: 2.7 (95% CI 1.8–3.9), SMR 85+: 1.1 (95% CI 0.6–1.9). Five years; SMR 65-74 yrs.: 10.1, (95% CI 7.7–13.0), SMR 75-84 yrs.: 7.4 (95% CI 5.8–9.3), SMR 85+: 3.8 (95% CI 2.8–5.0). Red line: SMR 1

Within 1 year after discharge, 8% (*n* = 55) of the discharged patients had died. The overall SMR and SMRs for subgroups of sex and age are shown in Fig. 2. The overall SMR was 1.9 (95% CI 1.5–2.5), indicating a nearly two-fold mortality rate in patients after burn injuries compared to the age and sex-matched general Dutch population. A non-significant trend in mortality rate for male and female at one-year after discharge was seen. Significant independent predictors of mortality at one-year post-discharge were a higher age (OR 1.1 95% CI 1.05–1.1), severe comorbidity (ASA ≥3) (OR 4.8 95% CI 2.3–9.7), and a non-home discharge location (OR 2.0, 95% CI 1.1–3.8) (Table 3). In addition, ICU stay and mechanical ventilation were more frequently reported. An increased revised Baux score and a longer length of stay were not associated with one-year mortality in multivariable regression (Table 3).

**Table 3:** Predictors of one-year mortality after discharge

	1 year mortality (***n*** = 681)		Univariable regression	Multivariable regression^**5**^
Characteristics	Survivors (*n* = 626)	Non-survivors (*n* = 55)	OR (95% CI)	OR (95% CI)
Sex, female	301 (48.1)	20 (36.4)	0.62 (0.35–1.0)	
Age, years	74.0 (69.0–80.0)	73.0 (69.0–80.0)	**1.09 (1.05–1.13)**^**3**^	**1.06 (1.02–1.11)**^**3**^
ASA score^1^				
1–2	396 (65.0)	11 (22.4)	1	
≥ 3	213 (35.0)	38 (77.6)	**6.42 (3.21–12.82)**^**3**^	**4.75 (2.32–9.72)**^**3**^
SES score^1^				
Other	430 (70.7)	36 (65.5)	1	
Low SES (1st quintile)	178 (29.3)	19 (34.5)	1.28 (0.71–2.28)	
TBSA burned				
< 5%	391 (62.5)	36 (65.5)	1	
5–19%	205 (32.7)	15 (27.3)	0.96 (0.43–1.49)	
≥ 20%	30 (4.8)	4 (7.3)	1.45 (0.48–4.34)	
Revised Baux Score	80.0 (73.5–88.0)	79.7 (73.0–87.6)	**1.05 (1.02–1.07)**^**3**^	–
Surgery				
No	163 (26.0)	13 (23.6)	1	
Yes	463 (74.0)	42 (76.4)	1.14 (0.60–2.17)	
TBSA excision (%)				
No surgery	163 (26.0)	13 (23.6)	1	
< 5%	338 (54.0)	30 (54.5)	1.11 (0.57–2.19)	
> 5%	125 (20.0)	12 (21.8)	1.20 (0.53–2.73)	
Length of stay (days)	14.5 (3.0–26.0)	14.0 (2.0–26.0)	**1.01 (1.00–1.02)**^**4**^	–
ICU stay				
No	507 (81.0)	42 (76.4)	1	
Yes	119 (19.0)	13 (23.6)	1.32 (0.69–2.53)	
Mechanical ventilation				
No	559 (89.3)	47 (85.5)	1	
Yes	67 (10.7)	8 (14.5)	1.42 (0.64–3.13)	
Discharge location^1^				
Home	457 (73.6)	25 (45.5)	1	
Other^*2*^	164 (26.4)	30 (54.5)	**3.34 (1.91–5.85)**^**3**^	**2.01 (1.06–3.79)**^**3**^

All data are presented as median (P25-P75) or as n (%)

^1^Missing: ASA *n* = 23, SES *n* = 18, Discharge location *n* = 5

^2^Including nursing facility, other hospital, psychiatric facility, revalidation facility

^3^*p* < 0.05

^4^Original value 1.009 (0.996–1.023) *p* < 0.20

^**5**^Nagelkerke r^2^ 0.18

### Mortality at five years post-discharge

After 5 years, 26% (*n* = 180/682) of the discharged patients had died (mortality rate of 668.0 per 10,000 person-years). The overall SMR was 1.4 (95% CI 1.2–1.6), again indicating excess mortality in patients after burn injuries compared to the general Dutch population (Fig. 2A). Figure 2B shows the SMRs at 5 years by age groups. The SMRs at 5 years were highest in patients aged 65–74 (SMRs 10.1, 95% CI 7.7–13.0) and in patients aged 75–80 years at 1 year (SMRs 2.7, 95% CI 1.8–3.9). Among the 180 patients deceased at 5 years, 125 diedbetween 1 year and 5 years after discharge. Older age was associated with an increased risk of dying (OR 1.1, 95% CI (1.0–1.1), as were severe comorbidity (OR 1.7, 95% CI 1.1–2.7, and a non-home discharge destination (OR 1.8, 95% CI 1.2–2.9) (Table 4).

**Table 4:** Predictors of mortality between one to five years after discharge

	1–5 years mortality (***n*** = 626)		Univariable regression	Multivariable regression^**6**^
Characteristics	Survivors (*n* = 501)	Non survivors (*n* = 125)	OR (95% CI)	OR (95% CI)
Sex, female	229 (45.7)	72 (57.6)	**1.61 (1.09–2.40)**^**3**^	–
Age, years	72.0 (69.0–79.0)	79.0 (71.0–85.0)	**1.08 (1.05–1.11)**^**3**^	**1.06 (1.03–1.09)**^**3**^
ASA score^1^				
1–2	228 (69.1)	58 (48.3)	1	1
≥ 3	151 (30.9)	62 (51.7)	**2.39 (1.59–3.59)**^**3**^	**1.72 (1.11–2.66)**^**3**^
SES score^1^				
Other	348 (71.2)	82 (68.9)	1	
Low SES (1st quintile)	141 (28.8)	37 (31.1)	1.11 (0.72–1.72)	–
TBSA burned				
< 5%	308 (61.5)	83 (66.4)	1	
5–19%	166 (33.1)	39 (31.2)	0.87 (0.57–1.33)	–
≥ 20%	27 (5.4)	3 (2.4)	0.41 (0.12–1.39)	–
Revised Baux Score	78.5 (72.2–86.0)	85.0 (77.2–93.0)	**1.04 (1.02–1.06)**^**3,5**^	**–**
Surgery				
No	139 (27.7)	24 (19.2)	1	
Yes	362 (72.3)	101 (80.8)	1.62 (0.99–2.63)	–
TBSA excision				
No surgery	139 (27.7)	24 (19.2)	1	1
< 5%	270 (53.9)	68 (54.4)	1.46 (0.88–2.43)	–
≥ 5%	92 (18.4)	33 (26.4)	2.08 (1.15–3.74)	1.35 (0.90–1.85)
Length of stay (days)	12.0 (2.0–25.0)	16.0 (5.0–27.5)	1.01 (1.00–1.016)^4^	–
ICU stay				
No	416 (83.0)	91 (72.8)	1	
Yes	85 (17.0)	34 (27.2)	**1.83 (1.16–2.90)**^**3**^	–
Mechanical ventilation				
No	456 (91.0)	103 (82.4)	1	
Yes	45 (9.0)	22 (17.6)	**2.16 (1.25–3.76)**^**3**^	**–**
Discharge location^1^				
Home	390 (78.2)	67 (54.9)	1	
Other^2^	109 (21.8)	55 (45.1)	**2.94 (1.94–4.45)**^**3**^	**1.84 (1.16–2.92)**^**3**^

All data are presented as median (P25-P75) or as n (%)

^1^Missing: ASA *n* = 23, SES *n* = 18, Discharge location *n* = 5

^2^Including nursing facility, other hospital, psychiatric facility, revalidation facility

^3^*p* < 0.05

^4^Original value 1.005 (0.995–1.016)

^5^In multivariate analysis revised Baux score was not included because of multicollinearity with age (Spearman correlation = 0.744) and mechanical ventilation was not included because of multicollinearity with ICU

^6^Nagelkerke r^2^ 0.19

Cox hazard analyses showed similar results. Up to 5 years after discharge, elderly burn patients with severe comorbidity had a more than a two-fold increased risk of dying compared to those with no or less severe comorbidity (HR 2.3, 95% CI 1.6–3.5). Furthermore, the relative risk of dying from discharge up to 5 years after discharge was predicted by a higher age (HR 1.1, 95% CI 1.0–1.1) and a non-home discharge location (HR 2.1, 95% CI 1.4–3.2).

## Discussion

This study assessed short-term and long-term mortality up to 5 years after burn injury and identified mortality predictors in elderly patients treated in specialized burn care. In total, 12% of admitted patients died in-hospital, 8% in the first 12 months after discharge and, at 5 years after discharge, up to 26% of the discharged patients had died. Overall, Standardized Mortality Ratios (SMR) at 1 year and 5 years after discharge were 1.9 (95%CI 1.5–2.5) and 1.4 (95% CI 1.2–1.6), respectively. With a remarkably high SMR in ‘young’ elderly (aged 65–74 years) at 5 years of 10.1 (95% CI 7.7–13.0). This study identified a higher age, severe comorbidity (ASA score ≥ 3, compared to ASA 1–2), and a non-home discharge location as independent predictors for mortality at 1 year and 5 years after discharge.

This study showed an increased mortality rate in elderly burn patients within the first 5 years after discharge compared to the general population. This is in line with previous studies that assessed long-term mortality, ranging from 5 years to 33 years after injury/discharge [5, 14, 17, 23]. However, other studies reported no differences in mortality rate [16] or even lower mortality rates in their burn population compared to the general population [24]. The lack of differences in mortality rates between the burn and non-burn population may be explained by the different comparison group that was used to compare the burn population, namely minor injury patients instead of the general population [16]. The lower mortality rate in the study of Nitzsche et al. can probably be explained by the difference in study populations of only severe burned patients (TBSA burned > 20%). In their study, survival bias is likely to play a role since only healthy and strong patients are likely to survive hospitalization and are, thus, likely to live longer after discharge [24].

When predictors of in-hospital mortality were compared to predictors of long-term mortality, we found that, while burn-specific variables such as TBSA burned and full-thickness burns and treatment-specific variables such as ICU admission and Revised Baux score were predictive of in-hospital mortality, they had no association with long-term mortality in our study. In addition, these predictors were highly predictive of in-hospital mortality (Nagelkere .58). However, long-term mortality prediction was less successful (Nagelkerke .19). This indicates that other variables that were not measured during this study might play a role in long-term mortality.

In general, explanations for the excess long-term mortality after burn injuries may be found in the systemic reactions and pathophysiological changes that remain after a burn [5, 14]. In our study, most patients had minor burns (TBSA< 5%), which might indicate that the systemic response is independent of burn size. Alternative explanations suggest that the increased mortality risk in elderly burn survivors could be linked to the inability of their immune system to overcome post-burn infections with an alternative inflammatory response. One study showed that elderly patients with burn injuries fail to initiate an appropriate inflammatory response during the acute phase after burn injury [25]. Furthermore, potential long-lasting systemic impacts on the heart and circulation play a role in the increased mortality in burn patients in general [9, 14]. It is likely, however, that this response is less applicable to our study population, as especially severe burn injuries are associated with a decreased cell-mediated immune response, increased stress hormones and sustained high levels of oxidative stress [5].

Severe comorbidity emerged as a prominent predictor of long-term mortality (OR 3.8, 95% CI 1.81–8.16). Thus, a higher incidence of comorbidity in the general burn population could explain the excess death apart from the potential long-term effects of the burn injury itself [14]. No literature is available on comorbidity severity in burn patients compared to the general population. So this would be an interesting subject to look into in the future. Thus, severe comorbidity seems not only related to a higher mortality risk for in-hospital patients, as has been shown by this study and in previous studies [26, 27] but might also an important predictor for long-term mortality.

Frailty could also play a role in the excess mortality of elderly patients with burn injuries [6, 15, 23, 28]. Elderly patients with severe comorbidity, or the inability to independently live at home after discharge, represent a group of dependent patients who are most likely to be frail. Frailty is “a complex age-related clinical condition characterized by a decline in physiological capacity across several organ system, with a resultant increased susceptibility to stressors” [29]. It is known that frailty can occur in burn injury survivors aged 50 years and over [30]. During hospitalization, frailty can increase in elderly patients with burn injuries [26], and frail patients have an increased in-hospital mortality rate [31, 32]. It could thus be possible that their frailty also plays a role in long-term mortality. Interestingly, in contrast to in-hospital mortality, where older patients from 75 years and over are more likely to die. Our study showed a nearly two-fold increased mortality rate in the younger elderly aged 65–74 years compared to their non-burn counterparts. Pre-injury frailty might explain the high SMR in relatively young elderly.

Previous literature suggests that frailty assessment on admission could realize a complete overview of the elderly burn patient that could lead to appropriate interventions during hospital stay [26]. Examples of frailty assessment used in burn care include The Clinical Frailty Scale (CFS) and Burn Frailty Index (BFI) [33, 34].

The most important clinical implication that can be drawn from this study is to focus on optimal and timely burn care for the elderly patient. The identified predictors for mortality in this study i.e., comorbidity, discharge destination, and age are not modifiable and, thus do not provide specific targets to focus on. In general, pre-injury health status should be assessed on admission, by screening for the presence of frailty at that time. Next, the systematic response to burn injuries should be minimized, for instance, with the application of adequate topical therapy such as Cerium Nitrate Silver Sulfadiazine, since this is thought to lower the toxic effect of the burn wounds [35, 36]. Alternatively, early excision and grafting of burn wounds might help in elderly patients with burn injuries. However, conflicting results have been published since some studies state that surgery in elderly patients can have adverse effects [37].

Finally, it is suggested that discharge location and the issues influencing them should be optimized prior to discharge to improve outcomes [23, 38]. In our study, patients with a non-home disposition, including nursing homes, rehabilitation centers, other hospitals, or hospices, had an increased mortality rate. Patients who cannot be discharged home, are not fit enough, and need additional rehabilitation or are in a terminal life phase. It could therefore be plausible that they are more likely to have an increased mortality rate compared to those who can be discharged to their homes. Discharge disposition has also been mentioned in previous literature as a predictor of mortality in patients with burn injury [23]. Pham et al. found a decrease in non-home discharge disposition in patients with aggressive inpatients rehabilitation, suggesting that early rehabilitation and mobilization might play a role in outcomes of the elderly burn population [38]. In our study population, inpatient rehabilitation was available for all patients. Early mobilization is an important factor in elderly hospitalized patients with hip fractures to prevent unnecessary loss of condition and return to usual activities as soon as possible [39–42]. The importance of early mobilization probably also applies to elderly patients with burn injuries and would be an interesting avenue to explore.

### Strengths and limitations

A significant strength of this study was the five-year follow-up period. Next to insight into the in-hospital mortality rate, our study created an extensive overview of long-term mortality. Furthermore, this multicentre national longitudinal cohort study covered a substantial period of 10 years and included all patients admitted to specialized burn care in the Netherlands from 2009 to 2018, covering a substantial period of 10 years.

The retrospective nature of this study has its limitations since the cause of death after discharge was unknown. Cause of death would have been valuable information, especially in long-term mortality, since it could give insight into the possible preventive measures that could be taken. Furthermore, a prospective study would have added to our knowledge of which specific organ systems were especially affected and might have shown long-term consequences of burn injuries.

This study did not have a control group of patients without burn injuries. However, using the SMRs, we could compare mortality rates to the general Dutch population. Nonetheless, it is debatablewhether our burn population is comparable to the general Dutch population given their high rate of severe comorbidity and frailty. It might thus be possible that the patients with burn injuries were sicker, frailer, and in a worse overall condition. Furthermore, previous literature has shown that both mental illness and substance abuse have a relatively high incidence in patients with burn injuries [14, 24]. It has been speculated that those factors might influence the early death of burn survivors [14]. Firchal et al. state that “an increased long-term mortality among adult burn survivors has been associated to new trauma or mental illness, rather than to the burn itself” [14]. We did not look at mental illness or substance abuse in this paper and encourage others to consider this possible predictor in future research.

In conclusion, this study adds to the scarce literature on especially long-term mortality of elderly patients with burn injury and the associated predictors. The increased improvement of in-hospital survival in the past decades mandates a more detailed understanding of predictors for long-term outcomes of burn patients. Independent predictors for long-term mortality in elderly patient with burn injuries at 5 years after discharge were higher age, severe comorbidity and a non-home discharge destination. As burn-specific variables were not found to be associated with long-term mortality, and it is thought that pre-existent comorbidity and frailty might play an important role, it is vital to prevent and target these factors to improve outcomes. Therefore, it must be stressed that alternative factors like frailty and frailty prevention and comorbidity monitoring must be examined.

## Data Availability

The datasets used and/or analysed during the current study available from the corresponding author on reasonable request.
